# Chronic Spontaneous Urticaria Severity Relating to TNF-Alpha Serum Concentration

**DOI:** 10.1155/drp/8853778

**Published:** 2025-04-19

**Authors:** Thai Van Thanh Le, Khanh Huy Mach, The Bich Thanh Vuong, Thien Tai Tran

**Affiliations:** ^1^Department of Dermatology and Skin Aesthetics, University Medical Center HCMC, Ho Chi Minh City 749000, Vietnam; ^2^Department of Dermatology, University of Medicine and Pharmacy at Ho Chi Minh City, Ho Chi Minh City 749000, Vietnam; ^3^Unit of Allergy and Clinical Immunology, University Medical Center HCMC, Ho Chi Minh City 749000, Vietnam

## Abstract

**Background:** Chronic spontaneous urticaria (CSU) is a prevalent skin disorder characterized by frequent recurrences. While its pathogenesis is closely associated with histamine and vascular activating mediators released by mast cells, some research suggests cytokines, notably tumor necrosis factor-alpha (TNF-alpha), could play a pivotal role in its pathology and symptom presentation.

**Objective:** This study evaluated serum levels of TNF-alpha in CSU patients and explored its correlation with clinical symptoms and severity at the University Medical Center at Ho Chi Minh City.

**Methods:** We enrolled 60 adult patients (age ≥ 18) with CSU, assessing their clinical symptoms using the UAS7 scoring system. TNF-alpha levels were determined utilizing the Avi Bion Human TNF-alpha kit. For comparative purposes, we also studied TNF-alpha levels in 30 healthy adult participants as a control group.

**Results:** The male-to-female ratio stood at roughly 1:2.3, and the median age was 36 (28–42). Notably, the mean serum concentrations of TNF-alpha in the patient group were considerably elevated compared to the control group (*p* < 0.001). A significant positive moderate correlation was found between serum concentrations of TNF-alpha and UAS7 score (*r* = 0.57; *p* < 0.001). Similarly, a notable positive moderate correlation between serum levels of TNF-alpha and pruritus scores was observed (*r* = 0.45; *p* < 0.001).

**Conclusion:** Serum levels of TNF-alpha are markedly increased in patients with CSU and show a moderate correlation with both UAS7 and pruritus scores. These findings suggest that TNF-alpha might play a potential role in the pathogenesis of CSU. However, further research involving a more extensive sample size is essential to draw definitive conclusions.

## 1. Introduction

Chronic spontaneous urticaria (CSU) is a relatively prevalent disorder with uniform distribution across national borders, age groups, and races, affecting up to 0.5%–1% of the global population [1]. This multifactorial disease, characterized by a high recurrence rate, often presents challenges in achieving sustainable symptom control. In its severe manifestation, patients' health and quality of life can markedly deteriorate without adequate management [1].

The pathogenesis of CSU is intricate, believed to be associated with histamine and other chemical mediators. Notably, auto-antibodies (anti-IgE) against diverse antigens, are thought to play a role in type I and IIb autoimmunity [2]. Furthermore, proinflammatory mediators, particularly cytokines, play a fundamental role in the pathomechanism of CSU [3].

Of the recognized proinflammatory mediators, tumor necrosis factor-alpha (TNF-alpha) is considered a central component in numerous autoimmune, neoplastic, and inflammatory ailments. TNF-alpha may contribute to CSU pathogenesis through three primary mechanisms. Initially, it activates and augments the expression of adhesion molecules on endothelial cell surfaces [4]. Subsequently, it incites mast cells to release histamine and other proinflammatory mediators, including leukotrienes, tryptases, prostaglandins, histamine, interleukin (IL)-1, IL-6, IL-8, and even TNF-alpha itself [4]. Finally, it is implicated in delayed cutaneous allergic responses such as specific IgE-dependent reactions [4]. Several investigations of serum concentrations of TNF-alpha in CSU patients have been undertaken [5, 6] highlighting a significant association between this cytokine and CSU symptoms. Concerning CSU treatment using anti-TNF-alpha drugs [7–9] preliminary clinical studies have reported encouraging outcomes, suggesting a potential new approach for disease management.

In this study, we measured serum concentrations of TNF-alpha in Vietnamese CSU patients to discern the relationship between this marker and the severity of symptoms.

## 2. Materials and Methods

### 2.1. Subjects

Patients with CSU who visited the University Medical Center at Ho Chi Minh City between August 2021 and October 2022 were considered. In all, 60 patients diagnosed with CSU based on clinical manifestations (in line with the EAACI/GA^2^LEN/EDF/WAO guideline) were selected on a consecutive basis. Written informed consent was obtained from all participants. Exclusions included patients diagnosed with acute urticaria (duration of urticaria < 6 weeks), urticarial vasculitis, systemic or autoimmune disorders, those on immunosuppressant drugs, and pregnant or lactating women. Thirty healthy adult volunteers from the hospital staff were invited to serve as the control group, with strict age and sex matching.

### 2.2. Study Method

#### 2.2.1. Study Design

This study had a case-control design. CSU patients meeting the inclusion criteria were provided a detailed explanation about the study's objectives and methodology. Opting of participation would not impact their treatment or benefits. Enrolled patients underwent clinical examination, interview, and evaluation using the Urticaria Activity Score over 7 days (UAS7) scale. Blood samples (3 mL venous blood) were collected from both patient and control groups to quantify TNF-alpha levels. These samples were first stored in test tubes with citrate. Subsequent quantification was conducted using the automated microplate processor EVOLIS Twin Plus system for the sandwich enzyme-linked immunosorbent assay (ELISA) performed with Alinity i (Abbott) analyzer.

#### 2.2.2. Assessment of UAS

UAS was used for assessment of disease activity in all patients. It was calculated as recommended by EAACI/GA2 LEN/EDF/WAO Guidelines. Weekly UAS was estimated according to the number of wheals and pruritus intensity, which had appeared during a week before the day of blood sampling. Patients were asked to document 24-h self-evaluation scores for 7 days applying the following scheme: no wheals = 0, 50 wheals/24 h = 3 and pruritus intensity: no = 0, mild = 1, moderate = 2, severe = 3. Weekly UAS equals the sum score of 7 consecutive days with a minimum score of 0 and a maximum score of 42. Consequently, weekly UAS were graded as follows: 0–14 (mild), 15–29 (moderate), and 30–42 (severe).

#### 2.2.3. Assessment of Pruritus Score

Daily UAS1 was used for assessment of pruritus score at blood sampling day. Patients were asked to document self-evaluation pruritus intensity for the day of TNF alpha blood testing applying the following scheme: pruritus intensity: no = 0, mild = 1, moderate = 2, severe = 3.

#### 2.2.4. Statistical Analysis

Data were processed using the Stata 14.2 software. Normal distribution of variables was checked using skewness and kurtosis test. Qualitative variables were expressed in terms of frequency and percentage, while quantitative variables were depicted as mean ± standard deviation for data with a normal distribution and as median accompanied by interquartile range for data that were not normally distributed. To evaluate the relationship between qualitative variables, the *χ*^2^ test was used. Comparisons between two groups were made using the Mann–Whitney *U* test, whereas the Kruskal–Wallis test followed by Dunn's multiple comparison test was employed to compare between more than two groups. Correlations between variables were ascertained using the Spearman's rank correlation coefficient, and *p* < 0.05 was considered statistically significant.

#### 2.2.5. Medical Ethics

The research received approval from the Ethics Committee in Biomedical Research of the University of Medicine and Pharmacy at Ho Chi Minh City.

## 3. Results

In total, 60 CSU patients and 30 healthy controls were included in the study. Among the CSU patients (60), 42 (70%) were female and 18 (30%) were male, yielding a ratio of 2.33:1. The average age of the patient group was 36 (28–42) years, ranging from 18 to 60 years (Table 1).

### 3.1. Duration of CSU and Symptoms

The clinical characteristics of patients with CSU including duration of CSU, duration of wheals, the presence of angioedema, family history, and UAS7 severity, alongside their corresponding TNF-alpha levels are summarized in Table 2.

### 3.2. Serum Concentrations of TNF-Alpha

The serum concentrations of TNF-alpha were significantly higher in the CSU group than in the control group (*p*=0.0002) (Table 3).

**Figure 1:**
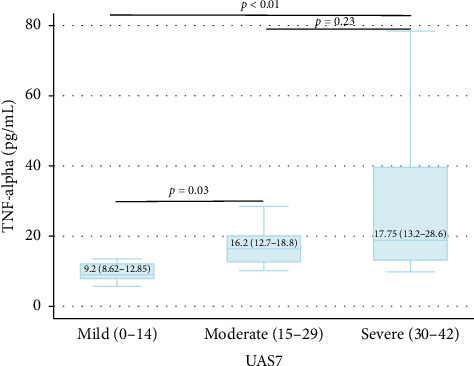
Statistical differences in TNF-alpha serum concentration among the mild, moderate, and severe CSU groups (assessed using the UAS7 scale). Kruskal–Wallis test: *p*=0.004. *p* values for comparisons between individual groups (Dunn's multiple comparison post hoc test) are shown.

**Figure 2:**
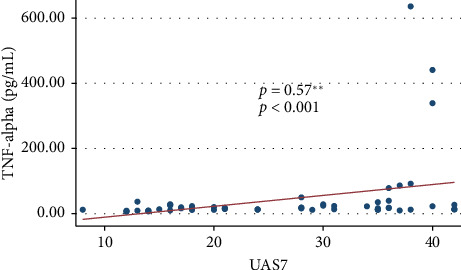
Correlation between serum concentrations of TNF-alpha and disease severity (assessed using the UAS7 scale). There was a moderate positive correlation between serum concentrations of TNF-alpha and disease severity (assessed using the UAS7 scale) (*ρ* = 0.57; *p* < 0.001).

As shown in Table 4, serum levels of TNF-alpha differed by severity of disease. There were statistically significant differences among the mild, moderate, and severe groups (Figure 1). In addition, we found a moderate positive correlation between serum concentrations of TNF-alpha and disease severity (Figure 2).

**Figure 3:**
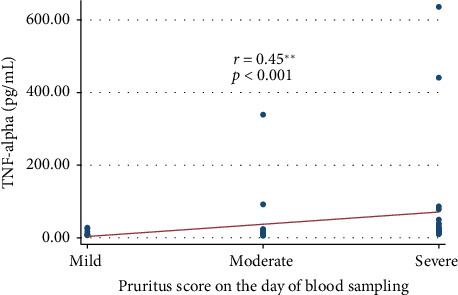
Correlation between serum concentrations of TNF-alpha and the pruritus score on the blood sampling day using the UAS7. There was a moderate positive correlation between serum concentrations of TNF-alpha and pruritus score on the day of blood sampling (assessed using the UAS7 scale) (*ρ* = 0.45; *p* < 0.001).

There was a statistical difference in the median serum concentrations of TNF-alpha between patients with no to moderate pruritus (0–3) and those with severe pruritus (4) (assessed using the UAS7 scale) (Table 5). In addition, we found a moderate positive correlation between serum concentrations of TNF-alpha and pruritus score on the day of blood sampling (Figure 3).

The diagnostic value of TNF-alpha serum concentration level for CSU was evaluated by an AUC-ROC analysis (Table 6).

## 4. Discussion

CSU is a prevalent skin condition that dermatologists encounter daily. The primary challenge is to achieve sustainable symptom control, which has yet to be fully addressed. The etiology of CSU is largely associated with histamine released by mast cells and other vascular activating mediators [1]. The involvement of peripheral leucocytes in CSU pathology is also under discussion [10], with IgG autoantibody against FcεRI and IgE reported in approximately one-third of CSU cases [2]. Recent research underscores the significant role cytokines play in CSU pathology [11–13].

In recent years, there has been heightened attention paid to serum concentrations of TNF-alpha in the CSU population. Our findings align with those of several previous studies that have reported elevated levels in CSU patients compared to healthy individuals [5, 6, 13] (Table 7). In addition, we found that levels significantly differed by disease severity, in line with two of those previous studies [5, 13] but not the third [6]. That third study had a sample size of 58 patients and used the UAS4 scale to assess severity, whereas we used the UAS7 scale. Given the potential variability in UAS clinical presentation over time, many authors have recommended using the sum of these two UAS scores to account for such fluctuations [14]. In CSU pathogenesis, TNF-alpha enhances the expression of endothelial adhesion molecules such as ECAM-1, VCAM-1, and E-selectin. This promotes leukocytes infiltration to the inflammation site, leading to dermal edema [10]. TNF-alpha also stimulates mast cells to release histamine [15] and contributes to mast cell-driven leukocyte infiltration linked to cutaneous late-phase reactions [16]. Thus, our research may highlight the potential utility of serum concentrations of TNF-alpha in gauging CSU severity in clinical scenarios, particularly given the moderate positive correlation between changes in TNF-alpha levels and CSU activity measured via the UAS7 scale.

We also identified a moderate positive correlation between TNF-alpha and the pruritus score, but not the wheal score on the blood sampling day. In CSU pathogenesis, there are two distinct types of wheals: an immediate wheal and a late wheal. Immediate wheals last for just 1 or 2 h and depend on swiftly released mediators such as histamine, which are quickly removed from the tissue site or neutralized. Conversely, late wheals emerge 4–8 h after the resolution of the immediate wheals, attributed to various factors including histamine, platelet activating factor, and products of arachidonic acid metabolism [17]. The expression of ICAM-1 and VCAM-1 on endothelial cells, which can be augmented by TNF-alpha, typically begins 4–6 h post induction, peaking at 16–24 h [10]. Similarly, the maximum count of leukocytes in inflammatory tissues due to TNF-alpha-induced infiltration is observed after 6–12 h [16]. In our study, 81.67% of CSU subjects had immediate wheals (< 4 h). Thus, there is no correlation between wheal scores on the day of blood sampling and serum concentrations of TNF-alpha, a pivotal cytokine in chronic inflammation.

Inflammation plays a crucial role in the pathogenesis of CSU. Research by Arzu Ataseven et al. revealed that the numbers of WBC, NEU, and PLT in the CSU group were significantly higher than those in healthy controls [18]. They also found a positive correlation between disease severity (assessed using the UAS7 scale) and the number of WBC and NEU [18]. These findings suggest a close relationship between these phenomena and increased serum concentration of TNF-alpha in CSU patients' blood. A study by Jingjing Wen et al. demonstrated a moderate positive correlation between TNF-alpha and WBC, while also indicating its significant association with various stages of leukemogenesis [19]. Moreover, other studies have shown that TNF-alpha triggers neutrophil recruitment by increasing ICAM-1 expression [20] and leads to platelet clumping and activation due to raised serum concentrations [21]. Based on these studies, it can be inferred that CSU is associated with increased inflammation and activation of inflammatory cells.

In the treatment of CSU, antihistamines remain the first option [22]. However, many cases do not respond to high-dose antihistamines, and in these situations, systemic treatment should be considered. Apart from antihistamines, other systemic treatments include oral corticosteroids and immunosuppressants such as azathioprine, methotrexate, oral tacrolimus, and mycophenolate mofetil [9]. Nevertheless, most of these treatments are not ideal for long-term control due to their side effects. It is worth mentioning that a study involving 20 patients explored monotherapy with TNF-alpha inhibitor (Adalimumab and Etanercept) for managing CSU symptoms revealed surprising results. Among 20 non-responsive patients who had received high-dose antihistamine and standard immunosuppressive therapies, complete or almost complete symptom resolution was observed in 60% of participants while partial improvement was seen in 15% [9]. Remarkably, there were also five significantly responsive CSU patients (8.3%) who did not benefit from omalizumab but showed positive responses to TNF-alpha inhibitors [9]. This indicates that there may be a specific group of patients who could derive benefits from TNF-alpha inhibitors even when omalizumab fails to control the disease's symptoms.

## 5. Limitations of the Study

Our study is unicentric with a small sample size and might not be representative of the entire CSU patient population, and each subgroup of mild, moderate, severe CSU also has a rather low number of patients.

## 6. Conclusion

We found elevated serum levels of TNF-alpha in CSU patients compared to healthy volunteers, pointing towards a potential role of this cytokine in CSU pathogenesis. Further comprehensive studies with larger sample sizes are needed to address remaining questions.

## Figures and Tables

**Table 1:** Age and sex distribution among cases and controls.

Characteristics	Cases (*n* = 60)	Control (*n* = 30)	*p*
Age	36 (28–42)	33.5 (26–40)	0.265^a^
Sex			
Male	18 (30%)	6 (20%)	0.312^b^
Female	42 (70%)	24 (80%)	

^a^Wilcoxon rank-sum test.

^b^Fisher's exact test.

**Table 2:** Clinical characteristics of CSU patients' group (*n* = 60).

Characteristics	Number of samples	Percentage (%)	TNF-alpha (pg/mL) Median (interquartile range)	*p*
*Duration of CSU*				
< 6 months	41	68.33	10.9 (7.26–15.8)	0.27^a^
> 6 months	19	31.67	18 (11.8–27.8)	

*Duration of wheals*				
< 4 h	49	81.67	15.6 (11.7–27)	0.25^a^
≥ 4 h	11	18.33	18 (13.5–25.2)	

*Angioedema*				
Yes	21	35	18 (13.5–25.2)	0.39^a^
No	39	65	10.9 (7.26–16.4)	

*Family history of CSU*				
Yes	16	26.67	21.65 (10.78–26.5)	0.34^a^
No	44	73.33	14.8 (12.4–22.7)	

*UAS7*				
Mild	8	13.33	9.2 (8.62–12.85)	0.004^b^
Moderate	26	43.33	16.2 (12.7–18.8)	
Severe	26	43.33	17.75 (13.2–28.6)	

^a^Wilcoxon signed rank test.

^b^The Kruskal–Wallis test.

**Table 3:** Serum levels of TNF-alpha of CSU patients compared to healthy controls.

	Number of samples	TNF-alpha (pg/mL) Median (interquartile range)	*p*
Cases	60	16.4 (12.6–22.95)	0.0002^a^
Controls	30	10.13 (7.22–15.3)	

^a^Wilcoxon rank-sum test.

**Table 4:** Correlation between TNF-alpha and the severity of CSU.

Severity	Number of samples	Median (interquartile range)	*p*
Mild	8	9.2 (8.62–12.85)	0.004^a^
Moderate	26	16.2 (12.7–18.8)	
Severe	26	17.75 (13.2–28.6)	

^a^The Kruskal–Wallis test. Measured using the Urticaria Activity Score over 7 days (UAS7) scale.

**Table 5:** Serum TNF-alpha of CSU patients with no to moderate pruritus and CSU patients with severe pruritus at blood sampling day (assessed using the UAS7 scale).

Pruritus score	Number of samples	Median (interquartile range)	*p*
No to moderate pruritus (0–3)	38	13.5 (11.5–18.8)	0.002^a^
Severe pruritus (4)	22	22.55 (14–39.6)	

^a^Wilcoxon signed rank test.

**Table 6:** TNF-alpha diagnostic ability for CSU.

	AUC–ROC	95% CI
TNF–alpha (pg/ML)	0.73	0.62–0.85

**Table 7:** Comparison of serum concentrations of TNF-alpha in patients and controls between studies.

Studies	Number of cases	Serum concentrations of TNF-alpha (pg/mL)		*p*
		Cases	Controls	
Present study	60	16.4 (12.6–22.95)	10.13 (7.22–15.3)	0.0002^a^
Atwa et al. [5]	75	17.93 ± 6.05	6.87 ± 3.73	0.004^b^
Grzanka et al. [6]	58	18.25 (17.04–19.62)	16.89 (16.45–18.40)	< 0.05^a^
Sharma et al. [13]	50	455.54 ± 253.54	8.498 ± 3.644	< 0.001^a^

^a^Wilcoxon rank-sum test.

^b^Student's *t*-test.

## Data Availability

Due to privacy and ethical concerns, neither the data nor the source of the data can be made available.
